# Granuloma annulare treated with certolizumab following the development of antidrug antibodies to adalimumab

**DOI:** 10.1016/j.jdcr.2025.03.028

**Published:** 2025-04-11

**Authors:** Ayelet I. Rubenstein, Jina Chung, Misha Rosenbach

**Affiliations:** a Perelman School of Medicine, University of Pennsylvania, Philadelphia, Pennsylvania; b Department of Dermatology, Hospital of the University of Pennsylvania, Philadelphia, Pennsylvania

**Keywords:** adalimumab, antidrug antibodies, certolizumab pegol, granuloma annulare, TNF-α inhibitor

## Introduction

Granuloma annulare (GA) is a chronic granulomatous skin disorder of unclear etiology characterized by annular plaques and erythematous papules.^1^ Although localized forms of GA may resolve spontaneously, the generalized variant is often chronic and resistant to therapy.^2^ First-line therapies often include antimalarials (eg, hydroxychloroquine), narrowband ultraviolet B, and intralesional triamcinolone, with adjuncts such as doxycycline or pentoxifylline for partial responders.^3^ Second-line treatments include tumor necrosis factor-alpha (TNF-α) inhibitors, which have been used off-label for recalcitrant GA and have shown efficacy in some cases.^3^

Several case reports and a single-center observational study have suggested that the TNF-α inhibitor adalimumab can be an effective and well-tolerated treatment for GA.^2^^,^^4^ However, limited data exist regarding options for patients with GA refractory to adalimumab. In addition, while the development of antidrug antibodies (ADA) has been documented in other diseases in response to TNF-α inhibitors,^5^ this phenomenon has not been described in GA to date. We now report for the first time on a patient with generalized granuloma annulare (GGA) who initially cleared on adalimumab, but experienced disease recurrence due to ADA development, in whom treatment with another TNF-α inhibitor, certolizumab, led to GGA remission.

## Case report

A 48-year-old female initially presented with a 5-month history of GGA involving the upper and lower extremities, abdomen, and back. Biopsy of flesh-colored papules on the right forearm showed an unremarkable epidermis and an interstitial and palisaded infiltrate composed of histiocytes, lymphocytes, and multinucleated giant cells with increased mucin deposition in the dermis. Initial therapies included a 3-antibiotic regimen (minocycline, ofloxacin, rifampin) without improvement; hydroxychloroquine, which provided partial response but was discontinued due to hair loss; and narrowband UVB phototherapy 3 times weekly, which stabilized her disease but it relapsed when frequency was reduced. Other therapies, including topical tofacitinib, intralesional triamcinolone, prednisone, dapsone, and methotrexate, were tested but provided limited benefit or were discontinued due to side effects. Two years after her initial development of GGA, adalimumab was initiated at a dose of 40 mg every other week, resulting in a significant improvement in erythema, induration, and body surface area involvement, and complete resolution of all lesions.

**Fig 1:**
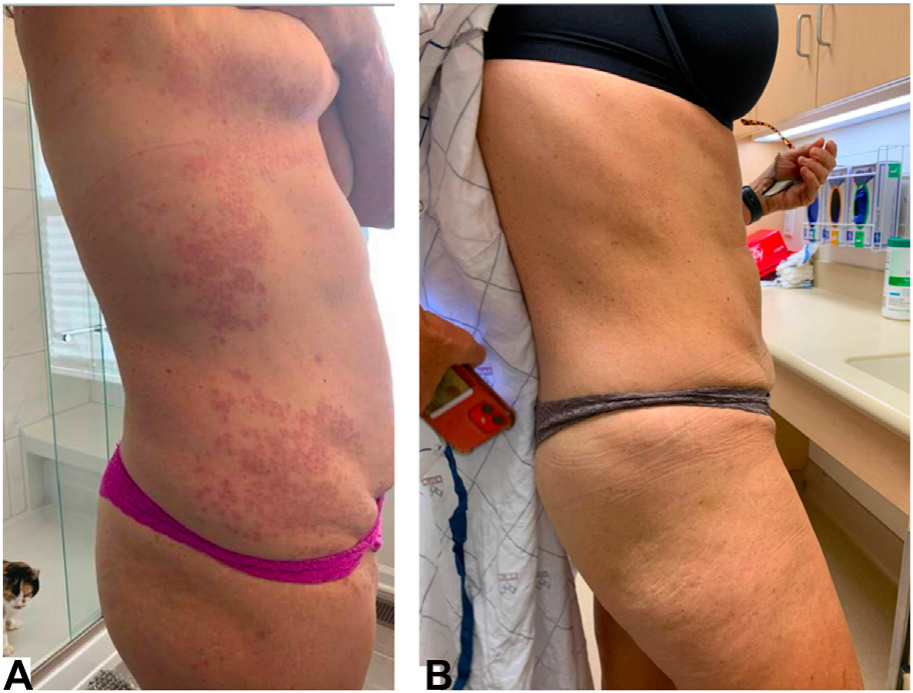
**A,** Generalized granuloma annulare (GGA) flare observed 3 months after discontinuation of adalimumab. The image shows a disseminated distribution of *pink-brown* plaques, many forming annular clusters, located on the trunk and inguinal folds. (Note: the background reflects a tele-dermatology submission by the patient during the COVID-19 pandemic). **B,** Resolution of GGA 14 months after initiating certolizumab.

One year after initiation of adalimumab, the patient was advised to taper the medication due to her complete response. However, the GGA recurred following the decrease in dosage. The patient self-restarted her prior dose of 40 mg every other week but continued to experience disease recurrence. Of note, the patient never used an adalimumab biosimilar during this period. Laboratory tests revealed low adalimumab drug levels of 1.5 μg/mL with high antiadalimumab antibody titers of 957 AU/mL (results of 25 or higher indicate detection of ADAs, with >300 indicating high titer). Adalimumab was stopped and hydroxychloroquine was briefly restarted at a dose of 200 mg twice daily, but the disease continued to flare. Physical exam at this time was notable for small red papules on extensor elbows and pink-brown plaques, many of which were annular, located on dorsal hands, feet, arms lateral trunk, inguinal folds, upper inner thighs, left medial leg, with ∼30% to 40% of BSA involved (Fig 1, *A*).

Given her previous response to adalimumab, she was transitioned to certolizumab pegol at a dose of 200 mg every 2 weeks. This resulted in a sustained and complete response with resolution of GGA (Fig 1, *B*). To date, she remains stable on this regimen with clear skin other than a faint erythematous annular plaque on her dorsal foot.

## Discussion

The management of GGA presents a significant therapeutic challenge due to its refractory nature and association with systemic conditions. The pathogenesis of GA remains incompletely understood but is thought to involve delayed-type hypersensitivity reactions mediated by helper T-cells and macrophages.^6^ Cytokines such as TNF-α and IL-1 play central roles in granuloma formation and tissue remodeling, providing a mechanistic basis for TNF inhibitors in treatment.

In this case, adalimumab initially provided a complete clinical response. However, ADA formation against adalimumab led to a loss of therapeutic effect, necessitating a switch to certolizumab pegol. Certolizumab, a TNF inhibitor with a distinct molecular structure and polyethylene glycol conjugation,^7^ demonstrated significant efficacy despite the prior failure of adalimumab. The phenomenon of ADAs is well-documented in other autoimmune conditions treated with biologics, such as rheumatoid arthritis, psoriasis, and Crohn disease. ADA development represents a significant limitation in biologic therapy, including TNF- α inhibitors.^5^

TNF-α inhibitors other than adalimumab such as infliximab and etanercept have been used in GA with varying degrees of success.^8^ However, to our knowledge no case of certolizumab use in GA has been reported to date. Compared to adalimumab, certolizumab lacks an Fc region, which reduces interactions with Fc receptors and minimizes the formation of ADAs.^9^ Additionally, its pegylation enhances stability and reduces immunogenicity, allowing for sustained therapeutic effects.^7^ Despite the potential advantages of certolizumab and its similar safety profile to adalimumab,^10^ adalimumab remains more commonly used due to its proven efficacy and extensive clinical experience.

This case highlights the therapeutic potential of certolizumab pegol in recalcitrant GGA, particularly following the failure of adalimumab due to ADA development. This novel use underscores the importance of considering alternative TNF inhibitors with distinct pharmacokinetic and immunogenic profiles in refractory cases. Our report highlights the need for further investigation into the immunogenicity of TNF inhibitors in dermatologic conditions. Future research should focus on mechanisms underlying GGA recurrence and strategies to mitigate ADA formation against TNF-inhibitors to ultimately achieve long-term treatment efficacy and improve outcomes for this challenging condition.

## Conflicts of interest

None disclosed.
